# Up-regulation of FGFBP1 signaling contributes to miR-146a-induced angiogenesis in human umbilical vein endothelial cells

**DOI:** 10.1038/srep25272

**Published:** 2016-04-28

**Authors:** Hua-yu Zhu, Wen-dong Bai, Jia-qi Liu, Zhao Zheng, Hao Guan, Qin Zhou, Lin-lin Su, Song-tao Xie, Yun-chuan Wang, Jun Li, Na Li, Yi-jie Zhang, Hong-tao Wang, Da-hai Hu

**Affiliations:** 1 Department of Burns and Cutaneous Surgery, Xijing Hospital, Fourth Military Medical University, Xi’an 710032, China; 2 Department of Hematology, Urumqi General Hospital of Chinese People’s Liberation Army, Urumqi 830000, China

## Abstract

Recent microRNA expression profiling studies have documented an up-regulation of miR-146a in several angiogenesis models. However, the underlying molecular mechanism of miR-146a in the angiogenic activity of endothelial cells has not been clearly elucidated. The present study was aimed to evaluate whether miR-146a promotes angiogenesis in HUVECs by increasing FGFBP1 expression via directly targeting CREB3L1. miR-146a was over expressed in HUVECs via lentiviral-miR-146a. Expression profiling analysis found miR-146a over expression resulted in up-regulation of angiogenesis and cytokine activity associated genes including FGF2. Further a combination of bioinformatics and experimental analyses demonstrated the CREB3L1 as a bona fide functional target of miR-146a during angiogenesis. Moreover, CREB3L1 inhibited luciferase expression from FGFBP1 promoter containing only CRE elements. Furthermore, CREB3L1 inhibited FGFBP1 expression by binding to two CRE-like sites located at approximately −1780–1777 and −868–865 bp relative to the FGFBP1 transcription start site. Additionally, ectopic expression of CREB3L1 decreased miR-146a-induced FGF2 secretion. These findings indicate that the miR-146a-CREB3L1-FGFBP1 signaling axis plays an important role in the regulation of angiogenesis in HUVECs and provides a potential therapeutic target for anti-angiogenic therapeutics.

Angiogenesis consists of the sprouting, migration, and remodeling of existing blood vessels, and plays important roles in various normal physiological processes^1^. However, deregulation of angiogenesis has been found in several pathological conditions and many human diseases^2^. Angiogenesis is a complicated multi-step process that is regulated by many potent angiogenic factors^3^. Basic fibroblast growth factor (FGF2) is one of the best-studied members of this family and has been shown to participate in a variety of biological programs, including embryonic development, tumorigenesis, and angiogenesis^4^,^5^. FGF2 promotes angiogenesis through stimulating the proliferation and migration of human umbilical endothelial cells (HUVECs)^6^,^7^. Since heparin-binding FGF2 is tightly bound to heparansulfate proteoglycans, and thereby trapped in the extracellular matrix, its release through the action of an FGF-binding protein (FGFBP1, also as known as BP1 and HBp17) is one of the critical steps in FGF2 activation^8^,^9^. Secreted FGFBP1 can serve as the angiogenic switch molecule that binds, mobilizes and activates the locally stored FGF2^9^,^10^. Toward cytokines stimuli, activated endothelial cells, especially HUVEC, are involved in the stepwise angiogenic process, such as degradation of the extracellular matrix, proliferation, migration and tube formation of endothelia cells^11^,^12^. However, the precise molecular mechanism of the regulation of HUVECs by FGFBP1/FGF2 during angiogenesis especially in solid tumors remains largely unknown.

CREB3L1 (cAMP responsive element-binding protein 3-like 1; also known as OASIS) is a member of the CREB3b ZIP transcription factor subfamily and was first identified in long-term cultured astrocytes and gliotic tissue^13^. CREB3L1 functions as a transcription factor that regulates target genes with important functions in many physiological processes^14^,^15^,^16^. Interestingly, CREB3L1 is down regulated in bladder cancer and acts as a tumor suppressor by directly suppressing tumor cell migration and colony formation^17^. Moreover, in an *in vivo* rat mammary tumor model, CREB3L1-expressing cells fail to develop metastases and experience impaired angiogenesis relative to CREB3L1-null cells, indicating its important role in suppressing tumorigenesis^18^. Nevertheless, the mechanism of the down regulation of CREB3L1 in cancer cells remains elusive.

MicroRNAs (miRNAs) are endogenous small non-coding RNA molecules capable of silencing protein coding genes by binding complementary sequences in 3′-untranslated regions (3′-UTR) of target mRNAs to induce their degradation or translational repression^19^. miRNAs can function as either oncogenes or tumor suppressors, and deregulated in most human cancers. miR-146a, first identified as an inflammation-related miRNA, has been shown to have angiogenic activity in the endothelial cells of a cancer cell model^11^,^20^. In addition, miR-146a plays a role in regulating angiogenesis in HUVECs during lipopolysaccharide (LPS) treatment^20^. However, the molecular mechanism by which miR-146a promotes angiogenesis has not been fully understood.

In this study, gene expression profile analysis was performed following over expression of miR-146a in HUVECs and found an up-regulation of genes associated with angiogenesis and cytokine activity. Further mechanistic study demonstrated that CREB3L1 was a direct target of miR-146a and negatively regulated the expression of FGFBP1 via binding a CRE-like site at FGFBP1 promoter.

## Materials and Methods

### Cell culture, cell lines and viral infection

HUVECs were obtained from the American Type Culture Collection (Manassas, VA, USA) and cultured in RPMI 1640 at 37 °C in a humidified atmosphere of 5% CO_2_. For lentivirus generation, a recombinant lentivirus carrying the human miR-146a precursor sequence was constructed by homologous recombination between the expression cosmid cassette and the parental virus genome in HEK293 cells. The recombinant lentivirus was then used to stably infect HUVECs as previously described^11^. HUVECs were infected with lentiviral vectors encoding shFGFBP1 and FGFBP1 cDNA as previously reported^21^. miR-146 overexpressing HUVECs were treated with FGF2 neutralizing antibody (F-5537, 8.8 μg/ml, Sigma, Deisenhofen, Germany) and FGFR inhibitor (NVP-BGJ398, 0.2 μM/ml, medchem express, Princeton, NJ, USA to evaluate the growth, migration and Tube formation effect, respectively.

### Microarray and bioinformatic analysis

Microarray analysis was performed to compare the gene expression profiles between HUVECs stably transfected with miR-146a and that with control lentivirus (Lv-control)^22^. Briefly, total RNA was isolated from the cells using the RNeasy kit (Qiagen) and analyzed in triplicate using the Human OneArray (Phalanx Biotech Group). Only genes with at least a 1.5-fold increase or decrease in expression and a significance of *P* < 0.05 were included in the final results. Gene ontology analysis and pathway enrichment analysis were performed using the DAVID website (http://david.abcc.ncifcrf.gov). miRwalk (http://www.umm.uni-heidelberg.de/apps/zmf/mirwalk/) was used to predict miR-146a targets^23^. Potential miR-146a-targeted genes associated with angiogenesis were analyzed and visualized using the DAVID website.

### Wound healing assay

HUVECs with different treatments were seeded in 6-well plates and incubated to 80% confluence. The cell monolayer was gently scraped with a 10 μl pipette tip, washed three times with PBS solution and incubated at 37 °C. Images were acquired using computer-assisted microscopy and the wound width was measured after 24 h. The migration area was determined using an Image-Pro Plus 6.0 system.

### Tube formation assay

Each well of pre-chilled 96-well plate was bottom-coated with 50 μl Matrigel (BD, San Diego, USA) and incubated at 37 °C for 30 min to polymerize. Then HUVECs with different treatments were seeded in each well and incubated at 37 °C of 5% CO_2_ for 12–18 h. Three microscope fields were selected at random and photographed. Tube forming ability was quantified by counting the total number of cell clusters (knots) and branches under a 10× objective and four different fields per well. The results are expressed as mean fold change of branching compared with the control groups. Each experiment was performed at least three times.

### Cell proliferation assay (MTT assay)

Cell proliferation was performed in triplicate by MTT assay according to the manufacture’s instruction (Sigma-Aldrich, St. Louis, MO, USA). Briefly, 1 × 10^5^ HUVECs/well were seeded in 96-well plate. Following treatment for the indicated time, 20 μl MTT was added to each well and incubated at 37 °C for 4 h. Supernatant was then removed and 150 μl dimethyl sulfoxide (DMSO) was added. After incubation at 37 °C for 15 min, the absorbency was measured with a micro ELISA reader (Bio phatometer, USA) at a wavelength of 570 nm. All assays were done in triplicate.

### Plasmid construction

The 3′UTR of CREB3L1 was amplified from human genomic DNA by PCR and cloned into a modified pGL3 luciferase vector (Promega, Madison, WI, USA) using the following primers: forward primer, 5′-TCTCCTAGGCCATGCCAAG-3′; and reverse primer, 5′-GTCCCTCTTTCCTGGGCCAG-3′. The PCR products with the appropriate primers generated inserts with point substitutions in the miRNA complementary sites to generate the pC3-CREB3L1-mut3′UTR vector as a mutant control^24^. Mut-CREB3L1-3′UTR vector was constructed by PCR with the appropriate primers to generate point substitutions in the miRNA complementary sites. The sequences of these constructs were verified by direct DNA sequencing.

The complete coding sequence of the CREB3L1 open reading frame was amplified from human genomic DNA by PCR and inserted into the pCDNA3 vector to get plasmid pC3-CREB3L1-wt using the following primers: forward primer, 5′-ATGGACGCCGTCTTGGAACCCTT-3′; and reverse primer, 5′-CTAGGAGAGTTTGATGGTGGTGTT-3′.

The human miR-146a precursor sequence was amplified from human genomic DNA by PCR and inserted into the pCDNA3 vector^11^. The 2-kb human FGFBP1 promoter (−2037 to +11 bp) was amplified from human genomic DNA by PCR and inserted into the pGL3-basic vector and designated as the pGL3-hFGFBP1 promoter. All plasmids for FGFBP1 promoter deletion constructs and mutants were generated by a PCR-based approach using the pGL3-FGFBP1 promoter (2 kb) as a template using the following primers: FGFBP1-wt forward, 5′-GTTTGGCAGCTCGGATCATGT-3′ (P1); reverse, 5′-CAGATCTTCATGGCTGCAGCT-3′ (P2); CRE1 (−2037–1521 bp) in FGFBP1 promoter was cloned with primers P1 and P3 5′-TGCCCTGATGGAATGCAGG-3′ (P3); CRE1 mut (−2037–1521 bp) in FGFBP1 promoter was cloned in two parts: CRE1 mut (−2037–1772 bp) with primers P1 and P4 5′-ACAACACTGTGGCCCTTTAC-3′, CRE1 mut (−1783–1521 bp) with primers P3 and P5 5′-AGGAGCTGTGAGTAAAGGGCCA-3′. Then the primers P1 and P3 were used to amplify CRE1 mut in the recombinant products contained −2037–1772 bp and −1783–1521 bp; CRE2 mut −1245–704 in FGFBP1 promoter recombinant plasmid was used in the same strategy, with following primers: 5′-GTTCATAGTTGTTTTTCTTA-3′ (P6); reverse, 5′-GAAGTAAGAGTTTAAGGAAG-3′ (P7) (for CRE2 (−1245–704)); P6 and 5′-AGTTCAGTGTGAAGGTGGT-3′ (P8) (for CRE2-mut (−1245–861)); P7 and reverse, 5′-TCAATAGGATGAACACCACCGGCA-3′ (P9) (for CRE2-mut (−1245–704)); Then the primers P6 and P7 were used to amplify CRE2 mut in the recombinant products contained −1245–861 bp and −1245–704 bp; All the constructs were verified by sequencing.

### *In vitro* luciferase assay

HEK-293 cells (50% confluence) in 48-well plates were transfected using Lipofectamine 2000 (Invitrogen, Carlsbad, CA, USA). The pC3-GFP-miR-146a or pC3-GFP (300 ng) along with a firefly luciferase reporter gene construct (100 ng) and a *Renilla* luciferase construct (10 ng; for normalization) were co-transfected into the cells. Firefly and *Renilla* luciferase activities were assessed using the Dual-Glo luciferase assay system (Promega, Madison, WI, USA) in accordance with the manufacturer’s instructions. Luminescence readings were acquired using a TD 20/20 luminometer (Turner Design Inc., Sunnyvale, CA, USA). Sample values were compared to the reference values of cells transfected with pC3-GFP. The experiments were performed in triplicate. The luciferase activities were measured as previously reported^25^.

### Chromatin immunoprecipitation assay (ChIP)

ChIP was performed as previously reported^25^. Briefly, protein–DNA crosslinking was initiated by directly adding formaldehyde to the culture medium at a final concentration of 1%, and cells were incubated for 15 min. Chromatin was prepared using a ChIP Assay Kit (Upstate Biotechnology) according to the manufacturer’s protocol. Equal amounts of chromatin from each sample were incubated overnight at 4 °C with 1 ml of anti-FLAG M2 (Sigma-Aldrich), anti-mouse IgG (Sigma-Aldrich), or anti-histone H3 (Santa Cruz Biotechnology) antibodies.

ChIP recovered DNA was then amplified by qPCR using primers covered a segment containing target region of the FGFBP1 promoter. Primers as follows: FGFBP1-1 (−1.7 kb) forward, 5′-GCAGACGGCAGTCACTAGG-3′; FGFBP1-1 reverse, 5′-CACTCTCGAAGACGCTGCT-3′; FGFBP1-2 (−0.8 kb) forward, 5′-GAACATTTGGGAAATCTCTTGC-3′; and FGFBP1-2 reverse, 5′-TGTGGCTCTGAAGGCAGTT-3′.

### Quantitative real-time PCR

Total RNA was extracted from cultured cells using TRIzol reagent (Invitrogen) according to the manufacturer’s instructions. cDNA was synthesized from 1 μg RNA using the PrimeScript RT Reagent Kit (Perfect Real Time; TaKaRa, Dalian, China) or the miScript II RT Kit (Qiagen, Germany). For detection of mRNA levels, we used fluorescence RT-qPCR with the following primers: FGFBP1 forward, 5′-CTTCACAGCAAAGTGGTCTCA-3′; FGFBP1 reverse, 5′-GACACAGGAAAATTCATGGTCCA-3′; FGF2 forward, 5′-AGAAGAGCGACCCTCACATCA-3′; FGF2 reverse, 5′-CGGTTAGCACACACTCCTTTG-3′; CREB3L1 forward, 5′-GCACCTGGACCACTTTACGG-3′; CREB3L1 reverse, 5′-AGCACAGGGTCATCAAAGAAG-3′; GAPDH forward, 5′-TCACCAGGGCTGCTTTTAAC-3′; GAPDH reverse, 5′-GACAAGCTTCCCGTTCTCAG-3′; miR-146a forward, 5′-UGAGAACUGAAUUCCAUGGGUU-3′; miR-146a reverse, Universal Primer (QIAGEN, Germany); U6-snRNA forward, RNU6B miScript Primer (QIAGEN, Germany); and U6-snRNA reverse, Universal Primer (QIAGEN, Germany). The RT-qPCR was analyzed using a Bio-Rad C1000 Thermal Cycler (Bio-Rad, USA) and the relative expression levels of miRNA and mRNA were calculated using the delta delta Ct method. GAPDH and U6-snRNA were used as an internal control for mRNA and miRNA, respectively.

### Enzyme-linked immunosorbent assay (ELISA)

The expression of FGFBP-1 and FGF2 was analyzed by ELISA as previously reported^26^. The cell growth-conditioned medium from the above experiments was collected and then analyzed for FGFBP-1 and FGF2. The concentrations were normalized to a control group. For each reaction in a 96-well plate, 100 ng of protein was used, and the ELISA was performed according to the manufacturer’s protocol (Promega, Madison, WI, USA). A standard curve was included in each experiment.

### Western blotting

Total protein was extracted from cells using lysis buffer containing 20 mM Tris-HCl (pH 7.4), 150 mM NaCl, 5 mM EDTA, 1% Triton-X 100, 1% DTT, and 1% protease inhibitor cocktail (Roche, Basel, Switzerland). Equal amounts of protein extracts (40 μg) were separated by 10% sodium dodecyl sulfate-polyacrylamide gel electrophoresis and transferred onto a PVDF membrane. Membranes were blocked with 5% w/v non-fat dry milk dissolved in Tris buffered saline plus Tween-20 (TBS-T; 0.1% Tween-20; pH 8.3) at room temperature for 1 h, then incubated with primary antibodies at 4 °C overnight. The following antibodies were used for Western blotting: mouse monoclonal anti-β-actin (A5441, Sigma-Aldrich, MO, USA), rabbit polyclonal anti-CREB3L1 (ab33051, Abcam, MA, USA), and rabbit polyclonal anti-FGFBP1 (sc-292235, Santa Cruz Biotechnology, TX, USA). After washing with TBS-T, membranes were incubated with horseradish peroxidase (HRP)-labeled secondary antibodies (Sigma, USA) for 1 h at room temperature. Immunobands were visualized using enhanced chemiluminescence (ECL) kit (GE Healthcare, Waukesha, WI, USA) according to manufacture’s instructions.

### Statistical analysis

All data were analyzed using SPSS 19.0 statistical software (version 19.0, SPSS, Chicago, IL, USA). Measurement data were expressed as mean ± SD. Comparisons were made by unpaired *t*-test and one-way ANOVA between groups. *P* < 0.05 was considered statistically significant.

## Results

### Over expression of miR-146a enhances angiogenic activity in HUVECs

**Figure 1:**
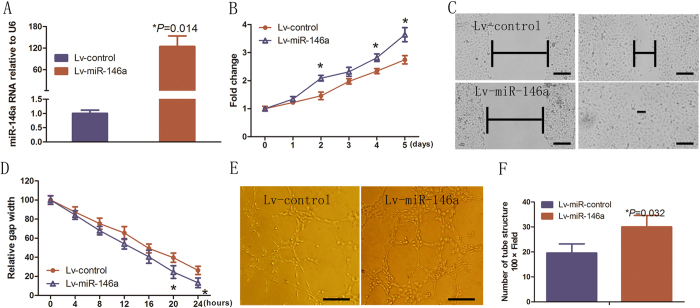
Over expression of miR-146a promoted the angiogenic phenotypes in HUVECs. (**A**) RT-qPCR analysis of miR-146a expression in HUVECs infected with Lv-control or Lv-miR-146a. Error bars represent mean ± SD from three experiments (n = 3); *P < 0.05. (**B**) Growth curves of HUVECs transduced with Lv-control or Lv-miR-146a. Error bars represent mean ± SD from three experiments (n = 3); *P < 0.05. (**C**) Scratch assay was conducted at the selected time points (per 4 h in 24 hs). Migration images of HUVECs infected with Lv-control or Lv-miR-146a in wound-healing assays. Images taken in 0 h and 24 h were shown. Scale bar: 100 μm. (**D**) Data represent the migration of the endothelial cell line in wound-healing assays for 0, 4, 8, 12, 16, 20, and 24 h. The scratch gap width at 0 h in each group was arbitrarily set at 1. Error bars represent mean ± SD from three experiments (n = 4); *P < 0.05, **P < 0.01. (**E**,**F**) Images and quantification of the tube formation assay of HUVECs transduced with Lv-control or Lv-miR-146a. Scale bar: 50 μm. Error bars represent mean ± SD from three experiments (n = 3); *P < 0.05, ANOVA (**A**,**B**) unpaired *t*-test (**D**,**F**).

To assess the potential biological function of miR-146a that might contribute to the biological behavior of HUVECs, a lentivirus-mediated delivery system was first used to stably express miR-146a in HUVECs. RT-qPCR showed that transduction of HUVECs with lentivirus-miR-146a (Lv-miR-146a) resulted in significant increase of miR-146a expression relative to control lentivirus (Lv-control)-infected HUVECs (*P* = 0.014; Fig. 1A). We next examined the proliferation, tube formation, and migration of HUVECs upon miR-146a over expression. MTT assay showed that miR-146a over expression significantly promoted the proliferation of HUVECs when compared to Lv-control (*P* < 0.05; Fig. 1B). Wound healing assay demonstrated miR-146a over expression increased the migratory ability of HUVECs (*P* < 0.05; Fig. 1C,D). In addition, tube formation assay revealed that miR-146a-overexpressing HUVECs formed more branches than that of Lv-control (*P* = 0.032; Fig. 1E,F). These results demonstrated that miR-146a enhanced the angiogenic activity of HUVECs.

### Over expression of miR-146a leads to upregulation of the expression of FGFBP1 and FGF2 in HUVECs

**Figure 2:**
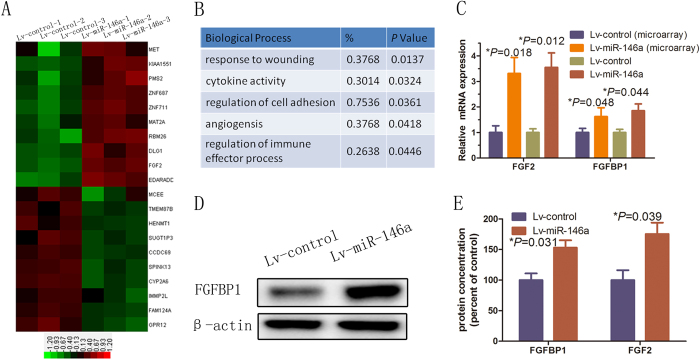
miR-146a promoted FGF2 and FGFBP1 expression. (**A**) Cluster analysis of mRNA expression profiles. Total RNA isolated from three biological replicates of Lv-miR-146a and Lv-control HUVECs was subjected to microarray analysis. The mRNA expression data were normalized to the average median of all genes present on the array. The mRNAs that were up-regulated at least 1.5-fold (red bars) or down regulated at least 2-fold (green bars) were considered for cluster analysis. (**B**) Gene Ontology classification of the predicted miR-146a target genes identified by integrating the results of four algorithms using the miRwalk website. (**C**) RT-qPCR was performed to determine FGF2 and FGFBP1 protein expression after infection of HUVECs with Lv-control or Lv-miR-146a. Error bars represent mean ± SD from three experiments (n = 3); *P < 0.05. (**D**) Western blot analysis of FGFBP1. (**E**) ELISA analyses of FGFBP1 and FGF2 protein expression. Error bars represent mean ± SD from three experiments (n = 3); *P < 0.05. ANOVA (**C**,**E**).

To explore the underlying mechanism of the promotion of angiogenesis of HUVECs by miR-146a, we performed the gene expression profiles of HUVECs over expressing miR-146a with that of control lentivirus (Lv-control)-infected HUVECs by a microarray analysis. Over expression of miR-146a led to significant alteration of 278 genes (Fig. 2A, Supplementary material 1). Further gene ontology analysis using DAVID bioinformatics resources revealed that the candidates were functionally enriched in several biological processes, including angiogenesis, cytokine activity, and immune effector processes (Fig. 2B). FGF2 promotes angiogenesis through stimulating the proliferation and migration of HUVECs^6^,^7^. miR-146a over expression resulted in significant up-regulation of FGF2 (Fig. 2B). Moreover, FGFBP1, which is the upstream molecule of FGF2 and functions as an angiogenic switch, was also increased by 1.5 fold following miR-146a over expression (data not shown). These results suggest that miR-146a may promote the angiogenesis of HUVECs by increasing FGFBP1/FGF2 signaling. To test this hypothesis, RT-qPCR assays was performed and found that the mRNA levels of both FGFBP1 (*P* = 0.044) and FGF2 (*P* = 0.012) were significantly increased in HUVECs over expressing miR-146a compared with those of the control (Fig. 2C). Further immunoblotting showed that the protein level of FGFBP1 in miR-146a-overexpressing HUVECs was significantly increased compared to that of control cells (Fig. 2D, SFig. 1A). Furthermore, the secreted levels of FGFBP1 (*P* = 0.031) and FGF2 (*P* = 0.039) were significantly increased in miR-146a-over expressing HUVECs compared to those of control cells (Fig. 2E). These results suggest the up-regulation of FGFBP1/FGF2 signaling may be one of the mechanisms of the promotion of angiogenesis by miR-146a.

### FGFBP1/FGF2 chemokine signaling promotes HUVECs proliferation, tube formation and migration

**Figure 3:**
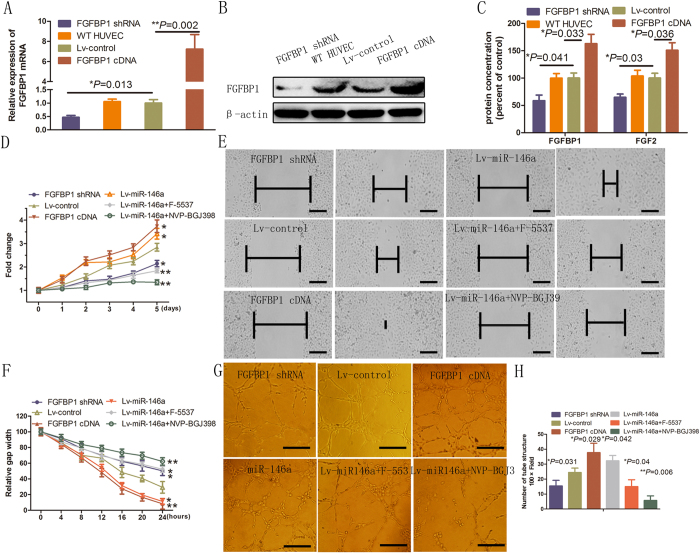
FGFBP1/FGF2 chemokine signaling promoted HUVECs proliferation, migration, and angiogenesis. (**A**,**B**) Transduction efficiency of FGFBP1 complementary DNA and shRNA in HUVECs as confirmed by RT-qPCR and Western blot analysis, respectively. Error bars represent mean ± SD from three experiments (n = 3); *P < 0.05. (**C**) FGFBP1 and FGF2 levels upon FGFBP1 cDNA and shRNA transfection in HUVECs. Error bars represent mean ± SD from three experiments (n = 3); *P < 0.05. (**D**) Growth curves of HUVECs transfected FGFBP1 cDNA or shRNA in a 24-well plate at the selective time points of 0, 1, 2, 3, 4 and 5 days. Error bars represent mean ± SD from three experiments (n = 3); *P < 0.05, **P < 0.01. (**E**) Representative scratch assay images in HUVECs. Images taken in 0 h and 24 h were shown. Scale bar: 100 μm. (**F**) Quantification of migration distances in scratch assay. Scratch gap width at 0 h in each group was arbitrarily set at 1. Error bars represent mean ± SD from three experiments (n = 4); **P < 0.05, **P < 0.01. Scale bar: 100 μm. (**G**) Tube formation assay images. Scale bar: 50 μm. (**H**) Quantification of the number of branches in the tube formation assay shown in (**G**). Error bars represent mean ± SD from three experiments (n = 3); *P < 0.05, ANOVA (**A**,**C**,**D**), unpaired *t*-test (**E**,**F**).

To explore the function of FGFBP1 in the angiogenic activity of HUVECs, HUVECs were transfected with a FGFBP1 short hairpin RNA (shRNA), which significantly reduced FGFBP1 at both the mRNA (*P* = 0.013; Fig. 3A) and protein (Fig. 3B) levels. Additionally, HUVECs were transfected with a plasmid carrying FGFBP1 cDNA to increase FGFBP1 expression. Transfection of HUVECs with FGFBP1 cDNA significantly increased FGFBP1 at both the mRNA (*P* = 0.002; Fig. 3A) and protein (Fig. 3B, SFig. 1B) levels. Interestingly, FGF2 protein secretion was increased by ectopic expression of FGFBP1 and decreased by FGFBP1 shRNA (Fig. 3C). We next examined the proliferation, tube formation, and migration of HUVECs upon either over expression or silencing of FGFBP1 in HUVECs. MTT assay showed that FGFBP1 over expression promoted while FGFBP1 shRNA inhibited the proliferation of HUVECs (*P* < 0.05; Fig. 3D). Similarly, scratch assay demonstrated that FGFBP1 over expression increased while FGFBP1 shRNA decreased the migratory ability of HUVECs (*P* < 0.05; Fig. 3E,F). In addition, tube formation assay raveled that FGFBP1 over expression stimulated (*P* = 0.034) while FGFBP1 shRNA reduced the formation of branches (*P* = 0.041; Fig. 3G,H). Taken together, these results demonstrated that FGFBP1/FGF2 chemokine signaling events are involved in the promotion of HUVEC proliferation, tube formation and migration.

### CREB3L1 is a direct target gene of miR-146a in HUVECs

**Figure 4:**
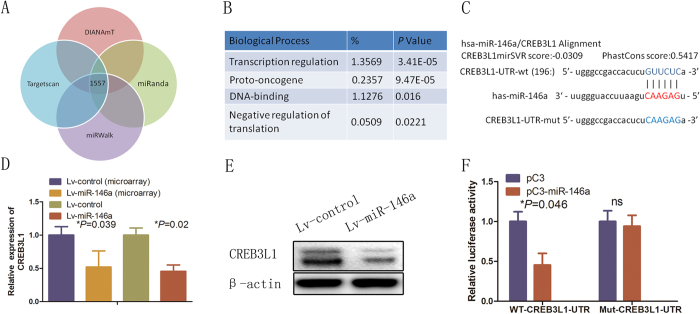
miR-146a directly targeted CREB3L1. (**A**) Gene Ontology classification of the predicted miR-146a target genes by integrating the results of four algorithms using the miRwalk website. (**B**) Gene Ontology enrichment analysis for 106 genes identified from the genes found in (**A**). (**C**) Schematic diagram of the miR-146a target site of human and other representative mammalian CREB3L1 3′UTRs. The wild-type 3′UTR of CREB3L1 and mutant 3′UTR sequences that abolished binding. (**D**) Reporter vectors containing the WT (wild-type) or MUT (mutant) CREB3L1 3′UTR were transfected along with Lv-control or Lv-miR-146a into HUVECs. Luciferase activity was measured in three independent experiments after 48 h of transfection and normalized to *Renilla* luciferase activity. Error bars represent mean ± SD from three experiments (n = 3); *P < 0.05. (**E**,**F**) RT-qPCR and Western blotting was performed to determine the CREB3L1 mRNA and protein expression, respectively, after infection with Lv-Luc or Lv-miR-146a. Error bars represent mean ± SD from three experiments (n = 3); *P < 0.05, ANOVA (**D**,**F**).

To explore the underlying molecular mechanism by which miR-146a over expression promotes the angiogenesis of HUVECs, we searched for potential miR-146a targets to predict in the whole human genome using the following bioinformatic miRNA target prediction tools: DIANAmT, miRanda, miRWalk and RNAhybrid (Fig. 4A). A total of 1,557 of miR-146a potential target genes were identified. Using DAVID Bioinformatics Resources, gene ontology analysis revealed that the candidate genes were functionally enriched in several biological processes (Fig. 4B). Several studies have demonstrated that most miRNAs regulate transcription factors at the mRNA level in angiogenesis^29^. Among these upregulated genes, CREB3L1 attracted our attention for two reasons. First, CREB3L1 has been associated with angiogenesis^17^ and its high expression suggests that angiogenesis events are involved in miR-146a-mediated promotion of HUVECs angiogenesis; second, it is one of the highest scoring target genes with a miR-146a-binding site in the 3′UTR of its mRNA. The CREB3L1 transcription factor was therefore focused to further narrow the candidates (Fig. 4C). Nevertheless, the mechanisms underlying miR-146a-upregulated CREB3L1 in HUVECs remain largely unknown.

Next, we performed RT-qPCR assays and found that the levels of CREB3L1 mRNA (*P* = 0.02; Fig. 4D) and protein (Fig. 4E, SFig. 1C) were significantly decreased in Lv-miR-146a-infected HUVECs relative to those in control Lv-Luc-infected HUVECs. Furthermore, CREB3L1 mRNA decreased by 0.58 fold in the microarray analysis (*not shown in the heatmap*). To further understand how miR-146a-overexpression inhibits CREB3L1 expression in HUVECs, we tested whether the 3′UTR of CREB3L1 is a direct target of miR-146a. We cloned the 3′UTR of CREB3L1 harboring the complementary sequence to the miR-146a seed sequence into a reporter plasmid vector. In parallel, the miR-146a seed sequence complementary site in the 3′UTR of the CREB3L1 in the same reporter plasmid was mutated (Fig. 4C). Transfection of HEK-293 cells with the CREB3L1-3′UTR construct along with miR-146a led to a significant decrease in luciferase activity relative to that of the control samples (*P* = 0.046; Fig. 4F). In contrast, the luciferase activity of cells transfected with the reporter vector containing a mutated 3′UTR of CREB3L1 was unaffected by simultaneous transfection of miR-146a (Fig. 4F). These results suggest that miR-146a directly binds to CREB3L1 mRNA and negatively regulates its stability and protein translation.

### CREB3L1 suppresses the gene transcription of FGFBP1 in HUVECs

**Figure 5:**
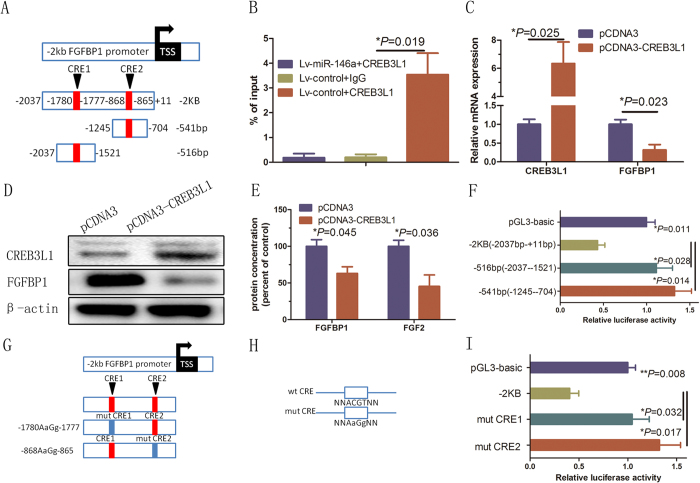
Functional analysis of CREB3L1-binding sites located in the human FGFBP1 promoter. (**A**) Schematic diagrams of the deleted reporter constructs from the 2-kb 5′-upstream promoter of the human FGFBP1 gene. Two putative CRE-like sites (containing an ACGT core) exist in the 2-kb FGFBP1 promoter region. (**B**) ChIP assay using an anti-CREB3L1 antibody or IgG. The immunoprecipitated DNA fragments and input were detected using PCR with specific primers at −2 kb. Error bars represent mean ± SD from three experiments (n = 3); *P < 0.05. (**C**–**E**) CREB3L1 over expression suppressed endogenous FGFBP1 expression in HUVECs. RT-qPCR and Western blot analyses of the relative mRNA and protein expression, respectively, in HUVECs infected with CREB3L1 or the control. Error bars represent mean ± SD from three experiments (n = 3); *P < 0.05. (**F**) Each deletion reporter vector and CREB3L1 expression vector was co-transfected. Reporter assays were performed 48 h after transfection. The reporter activities significantly decreased in cells transfected with the 500-bp construct, suggesting that CREB3L1 transcriptionally inhibits FGFBP1. Error bars represent mean ± SD from three experiments (n = 3); *P < 0.05. (**G**) Schematic diagrams of the mutated reporter constructs. (**H**) The panel showed schematic representations of the wild-type CRE-like site containing an ACGT core and the mutated CRE-like sites containing an AAGG core. (**I**) Reporter assays using HUVECs. Each mutated reporter vector and the CREB3L1 expression vector were co-transfected. Reporter assays were performed 48 h after transfection. The reporter activities significantly decreased in cells transfected with the mutated CRE-like site constructs. Error bars represent mean ± SD from three experiments (n = 3); *P < 0.05, **P < 0.01, ANOVA (**B**,**C**,**E**,**F**,**I**).

The potential mechanism of the regulation of FGFBP1/FGF2 signaling by miR-146a-CREB3L1 axis in HUVECs was then explored. DNA sequence analysis revealed the presence of two CRE-like sites (containing an ACGT core) in the FGFBP1 promoter (Fig. 5A). Within the 2-kb promoter of the FGFBP1 gene, specific CREB3L1-binding sites were identified, suggesting that CREB3L1 might function as a transcriptional suppressor that binds to the FGFBP1 promoter region. To validate this prediction, a 2-kb FGFBP1 promoter sequence (−2037 to +11 bp from the human FGFBP1 transcriptional start site) was cloned into the pGL3-basic reporter plasmid (pGL3-hFGFBP1 promoter, 2 kb). ChIP demonstrated that the CREB3L1 antibody specifically pulled down the FGFBP1 promoter in HUVECs (*P* = 0.019, Fig. 5B).

To investigate the regulation of FGFBP1 by CREB3L1 in HUVECs, we examined FGFBP1 expression levels in HUVECs infected with a vector stably expressing the CREB3L1 (*P* = 0.025) (Fig. 5C,D). The FGFBP1 mRNA (*P* = 0.023; Fig. 5C) and protein (Fig. 5D, SFig. 1D) levels were significantly decreased in the CREB3L1-infected cells. Furthermore, the secretion of FGFBP1 (*P* = 0.045) and FGF2 (*P* = 0.036) was reduced in the CREB3L1-infected cells (Fig. 5E). We further constructed truncated reporter genes from the original 2-kb human FGFBP1 promoter that contained two CRE-like sites (Fig. 5A). The luciferase activities in HUVECs transfected with the 500-bp (−1780 to −1777 bp and −868 to −865 bp) reporter construct were dramatically reduced (*P* = 0.028 and *P* = 0.014; Fig. 5F).

To test that the CRE-like sites interact with CREB3L1, we generated mutated reporter constructs that substituted the ACGT core sequence with an AAGG sequence in each CRE-like site (Fig. 5G,H). The reporter activities in cells transfected with the construct containing mutated CRE-like sites 1 and 2 were significantly increased, whereas the activities in cells transfected with the other mutated constructs were enhanced by CREB3L1 (*P* = 0.032 and *P* = 0.017; Fig. 5I). As mutation of CRE-like sites 1 and 2 at FGFBP1 promoter may result in loss of the suppression by CREB3L1, these results indicated that CREB3L1 specifically acts on CRE-like sites 1 and 2 in the human FGFBP1 promoter to inhibit its transcription.

### CREB3L1 over expression inhibits miR-146a-induced FGF signaling in HUVECs

**Figure 6:**
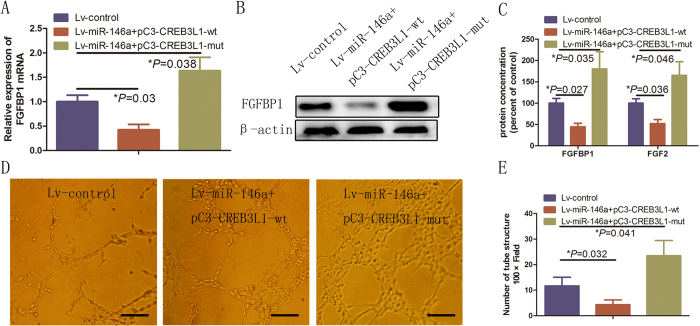
CREB3L1 was a mediator in miR-146a over expression-induced FGFB1 and FGF2 expression. (**A**,**B**) RT-qPCR and Western blot analysis of FGFBP1 when CREB3L1 was up-regulated in HUVECs stably over expressing miR-146a. Error bars represent mean ± SD from three experiments (n = 3); *P < 0.05. (**C**) ELISA demonstrating the amount of FGFBP1 and FGF2 released from cultured HUVECs under the same treatment. Error bars represent mean ± SD from three experiments (n = 3); *P < 0.05. (**D**,**E**) Images and quantification of HUVECs tube formation following transfection of wild type (WT) and mutant of CREB3L1 in HUVECs over expression miR-146a. Error bars represent mean ± SD from three experiments (n = 3); *P < 0.05. Scale bar: 50 μm. ANOVA (**A**,**C**), unpaired *t*-test (**E**).

Our previous observations showed that CREB3L1 is a functional target of miR-146a and a transcriptional repressor of FGFBP1, which promotes angiogenesis, suggesting that CREB3L1 over expression may attenuate the angiogenesis induced by miR-146a over expression. This hypothesis was tested by transfecting exogenous CREB3L1 cDNA into miR-146a-transfected HUVECs. CREB3L1 over expression significantly abolished the induction of FGFBP1 mRNA (*P* = 0.03; Fig. 6A) and protein (Fig. 6B, SFig. 1E) in miR-146a-overexpressing HUVECs and prevented the secretion of FGFBP1 protein into the cell culture medium (Fig. 6C). Consistent with the key function of the CREB3L1 transcription factor in angiogenesis, transfection of the constructs containing the mutated CRE-like sites prevented the induction of FGFBP1 (*P* = 0.027; Fig. 6A–C) and FGF2 expression in miR-146a-over expressing HUVECs (*P* = 0.036; Fig. 6C). Moreover, CREB3L1-mutation increased FGFBP1 and FGF2 mRNA and protein levels in miR-146a over expressed HUVECs (Fig. 6A–C). Finally, we assessed whether CREB3L1 expression could regulate angiogenesis in miR-146a over expressed HUVECs. The data showed that the wide type CREB3L1 suppressed the effects of miR-146a over expression on the promotion of angiogenesis (*P* = 0.032; Fig. 6D,E), while miR-146a-induced angiogenesis was increased by CREB3L1 with mutated binding sites of FGFBP1 promoter (*P* = 0.041; Fig. 6D,E). Taken together, these results indicated that CREB3L1 over expression abrogates miR-146a over expression-induced angiogenesis, suggesting that CREB3L1 is a functional mediator of miR-146a activity in the regulation of angiogenesis in HUVECs.

## Discussion

In the present study, we found that over expression of miR-146a promoted angiogenesis in HUVECs, accompanied with an increased expression of FGFBP1 and FGF2. Mechanistically, it was demonstrated that miR-146a directly targeted CREB3L1, which in turn repressed the gene transcription of FGFBP1. These findings suggest that miR-146a enhances angiogenesis in HUVECs through promoting the expression of FGFBP1 and FGF2 via directly targeting CREB3L1.

Previous studies have shown that miR-146a is involved in the regulation of the innate immune response^30^,^31^. It has been recently found that miR-146a plays an important role in tumorigenesis^32^,^33^. Sun *et al.* found that miR-146a functions as a tumor suppressor in prostate cancer by suppressing growth, migration and invasion^34^. Moreover, clinicopathological data have demonstrated that miR-146a expression is lower in hepatocellular carcinoma tissues than in adjacent non-cancerous hepatic tissues^35^,^36^. In contrast, a recent report has indicated that miR-146a may function as an oncogene in the development of acute promyelocytic leukemia (APL), and is a novel prognostic biomarker in APL^34^. Nonetheless, the roles of miR-146a in regulating vascular proliferation and angiogenesis and the underlying molecular mechanism have not been fully elucidated. The GO analysis of mRNA array data indicated that miR-146a up-regulation might enhance the angiogenic activity of endothelial cells. This finding was consistent with previously reported data in other cohorts^37^, further confirming a biological role of miR-146a in the development of angiogenesis. However, the underlying mechanism of miR-146a in promoting angiogenesis in HUVECs remains unclear.

Previous studies have found miR-146a target several signaling pathways including EGF and WASF2 pancreatic cancer, gastric cancer, and squamous cell carcinoma^38^,^39^,^40^. Accumulating evidence demonstrates that miR-146a plays an important role in the biological processes in endothelial cells^11^,^41^, but the mechanism remains elusive. CREB3L1 is a transcription factor that regulates the expression of many genes, including ER chaperones such as GRP78^42^. Several pieces of evidence have demonstrated the loss of CREB3L1 expression in malignant cancer cells and that the maintenance of CREB3L1 expression could potentially suppress tumorigenesis^16^,^42^. The bioinformatics analysis and luciferase assays showed that CREB3L1 is a bonafide target of miR-146a during HUVEC angiogenesis. These facts suggest that miR-146a may promote tumorigenesis and angiogenesis at least in part by targeting CREB3L1 in endothelial cells.,

FGF2 is a pro-angiogenic factor that is involved in the pathophysiology of several ocular diseases involving neovascularization, especially in HUVECs^43^,^44^. Secreted FGFBP1 acts as a chaperone molecule and binds to FGF2 in a reversible, noncovalent manner; it also positively modulates the biological activities of autocrine FGF2, thus supporting tumor growth and angiogenesis^8^,^10^,^45^. Therefore, the identification of angiogenic factor regulation is crucial for understanding the complete function of FGFBP1 in cells and for identifying the mechanisms of its control over cellular processes and angiogenic development^46^. Previous studies have demonstrated CREB3L1 is a transcriptional activator^47^,^48^. In the present study, we demonstrated that CREB3L1 over expression in HUVECs reduced FGFBP1 mRNA and protein levels, and increased the expression of a reporter gene carrying the 2-kb 5′-upstream promoter region of the FGFBP1 gene. Moreover, CREB3L1 directly bound to the promoter region containing CRE-like sites 1 and 2. These findings suggest that CREB3L1 inhibits the expression of FGFBP1 by directly binding to its promoter region in HUVECs, which is supported by the GEO database in MDA-MB-435 (GSM1252272) and LN4D6 (GSM1252957) cells.

In summary, the results demonstrated that CREB3L1 is a mediator of miR-146a and FGFBP1 in angiogenesis of HUVECs, suggesting that targeting miR-146a-CREB3L1-FGFBP1 signaling axis is a potential therapeutic strategy for anti-angiogenic therapeutics. However, future studies are needed to further investigate the role of miR-146a in promoting angiogenesis *in vivo*.

## Additional Information

**How to cite this article**: Zhu, H.-y. *et al.* Up-regulation of FGFBP1 signaling contributes to miR-146a-induced angiogenesis in human umbilical vein endothelial cells. *Sci. Rep.* **6**, 25272; doi: 10.1038/srep25272 (2016).

## Supplementary Material

Supplementary Information

Supplementary Dataset
