# Supplementary material for: Up-regulation of FGFBP1 signaling contributes to miR-146a-induced angiogenesis in human umbilical vein endothelial cells
Source: Sci Rep. 2016 Apr 28;6:25272. doi: 10.1038/srep25272 (PMC4848533; doi:10.1038/srep25272)
Supplement: Supplementary Information [file srep25272-s1.doc]

**Up-regulation of FGFBP1 signaling contributes to miR-146a-induced angiogenesis in human umbilical vein endothelial cells**

Hua-yu Zhu^1,§^, Wen-dong Bai^2,§^, Jia-qi Liu^1,§^, Zhao Zheng^1^, Hao Guan^1^, Qin Zhou^1^, Lin-lin Su^1^, Song-tao Xie^1^, Yun-chuan Wang^1^, Jun Li^1^, Na Li^1^, Yi-jie Zhang^1^, Hong-tao Wang^1,*^, Da-hai Hu^1,*^

^1^Department of Burns and Cutaneous Surgery, Xijing Hospital, Fourth Military Medical University, Xi’an 710032, China.

^2^Department of Hematology, Urumqi General Hospital of Chinese People’s Liberation Army, Urumqi 830000, China.

**Supplementary Figure Legends:**


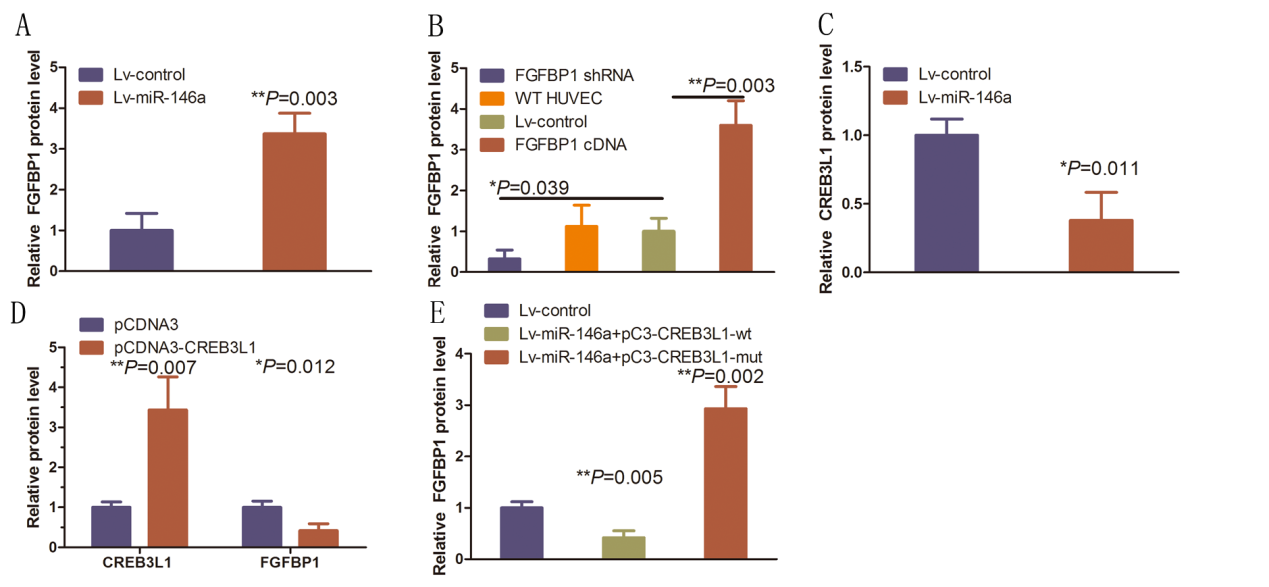


SFig. 1 (A) Protein expression of FGFBP1 in miR-146a overexpressing HUVECs. Values shown are the mean±SD for each group. (B) Protein expression of FGFBP1 in FGFBP1 knockdown, overexpression and wild type HUVECs. (C) Protein expression of CREB3L1 in miR-146a overexpressing HUVECs. (D) Protein expression of CREB3L1 and FGFBP1 in CREB3L1 overexpressing HUVECs. (E) Protein expression of FGFBP1 in each group. The protein level after normalization against its corresponding β -actin was arbitrarily set at 1 (n=3), **P*<0.05, ***P*<0.01.
